# Resistance valves in circulatory loops have a significant impact on *in vitro* evaluation of blood damage caused by blood pumps: a computational study

**DOI:** 10.3389/fphys.2023.1287207

**Published:** 2023-11-30

**Authors:** Peng Wu, Yuqiao Bai, Guanting Du, Liudi Zhang, Xiangyu Zhao

**Affiliations:** ^1^ Jiangsu Key Laboratory for Design and Manufacture of Micro-Nano Biomedical Instruments, School of Mechanical Engineering, Southeast University, Nanjing, China; ^2^ Artificial Organ Technology Laboratory, School of Mechanical and Electrical Engineering, Soochow University, Suzhou, China

**Keywords:** circulatory loop, blood pump, resistance valve, hemolysis, platelet activation, computational fluid dynamics

## Abstract

**Background:** Hemolysis and its complications are major concerns during the clinical application of blood pumps. *In-vitro* circulatory testing loops have been employed as the key procedure to evaluate the hemolytic and thrombogenic performance of blood pumps during the development phase and before preclinical *in-vivo* animal studies. Except for the blood damage induced by the pump under test, blood damage induced by loop components such as the resistance valve may affect the accuracy, reproducibility, and intercomparability of test results.

**Methods:** This study quantitatively investigated the impact of the resistance valve on *in vitro* evaluation of blood damage caused by blood pumps under different operating points. A series of idealized tubing models under the resistance valve with different openings were created. Three pumps - the FDA benchmark pump, the HeartMate 3 LVAD, and the CH-VAD - were involved in hypothetical tests. Eight operating points were chosen to cover a relatively wide spectrum of testing scenarios. Computational fluid dynamics (CFD) simulations of the tubing and pump models were conducted at the same operating points.

**Results and Conclusion:** Overall, hemolysis and platelet activation induced by a typical resistance valve are equivalent to 17%–45% and 14%–60%, respectively, of those induced by the pump itself. Both ratios varied greatly with flow rate, valve opening and pump models. Differences in blood damage levels between different blood pumps or working conditions can be attenuated by up to 45%. Thus, hemolysis and platelet activation induced by the resistance valve significantly affect the accuracy of *in-vitro* hemocompatibility evaluations of blood pumps. A more accurate and credible method for hemocompatibility evaluations of blood pumps will benefit from these findings.

## 1 Introduction

For end-stage heart failure, blood pumps have gradually become the bridge to, or even replaced heart transplantation (Magruder et al., 2017; Shahreyar et al., 2018; Rogers et al., 2019; Virani et al., 2021). In blood pumps, complex geometric structures and movements of the impeller induce non-physiological stresses which can be up to two orders of magnitude than physiological ones (Garon and Farinas, 2004; Fraser et al., 2011; Mei et al., 2022), increasing the risk of blood damage, including hemolysis, that is, the release of hemoglobin (Hb) into plasma. Device-related adverse events (AEs), such as bleeding, stroke, and thrombosis, are therefore often reported after implantation (Birschmann et al., 2014; Gurbel et al., 2018), limiting the application of these devices.

Thus, evaluation of hemocompatibility of blood pumps is required not only during the development phase, but also for regulatory approval before human clinical use. In a typical development process of blood pumps, computational fluid dynamics (CFD) combined with *in-vitro* experiments are usually employed to estimate the hydrodynamic and hemolytic performance to reduce the cost (Huo et al., 2021; Wu et al., 2021). Hydraulic tests are needed to validate the hydrodynamic performance, while *in-vitro* blood circulatory testing is a crucial step to assess the hemocompatibility of blood pumps before preclinical *in-vivo* animal studies (Food and Drug Administration Center for Devices and Radiological Health, 2010). A typical *in-vitro* blood circulatory loop includes tubing, connectors, sampling ports, reservoir, and resistance valve, in addition to the blood pump under test. So far, a wide variety of *in-vitro* test loops have been proposed and put into use, and protocols such as ASTM F1841-19e1 (American Society for Testing and Materials, 2021) provided some recommendations for *in vitro* evaluation of blood pumps; but a specific guidance is still lacked on how to choose various components and build the *in-vitro* blood circulatory loop.

Except for the blood damage induced by the pump itself, other loop components may also cause blood damage, which could affect the credibility of experimental data. Studies suggest that loop geometry (Kazui et al., 2016; Chiu et al., 2017), connectors (Fuchs et al., 2018; Li et al., 2020), and air bubbles (Mullins and Bruns, 2017) can affect blood damage of the device under test, limiting the reproducibility of tests, as well as the intercomparability between tests conducted on different loop configurations or by different labs. Olia et al. (Olia et al., 2016) developed a reusable, compliant, and small volume blood reservoir for *in-vitro* hemolysis testing rigid, which could minimize resistance, promote mixing, prevent bag collapse, and allow for complete de-airing. Li et al. (Li et al., 2020) found that loops with more components (5-connector loop and 90° T-connector) showed 63% and 128% higher platelet activation levels, respectively, *versus* those with fewer (0-connector) and 90° heat-bend loops. These findings underscored careful consideration of all component elements in the loop.

The resistance valve is among the key components of the *in-vitro* blood circulatory loop. The resistance valve is applied to the tubing to locally form a narrow fluid path, thus creating adequate back pressure on the pump. Meanwhile, the sudden contraction and expansion of the fluid path may lead to a drastic increase in shear stresses and secondary flow in the blood flow, thus adding significantly to the overall blood damage. As observed in (Down et al., 2011; Herbertson et al., 2014), sudden contraction and expansion significantly increased hemolysis, with the sudden contraction causing the most blood damage. As Bluestein and Mockros reported (Bluestein and Mockros, 1969), hemolysis is positively associated with pressure head. The pressure head of the blood pump under test is mainly balanced by the resistance valve. The blood damage induced by the valve is presumably comparable with the hemolysis of the pump itself.

The hemolysis induced by the resistance valve was already recognized by some researchers. James et al. (James et al., 2003) applied a long clamp over a tubing of 10 cm length, to minimize the hemolytic potential caused by local narrowing of the tubing. However, according to Schima et al. (Schima et al., 1993), compared with clamps (throttles), using 4.5 m-long-tubings as resistance shows little difference in hemolytic potential, indicating that longer exposure time also increases the risk of hemolysis.

The hemolysis and platelet activation induced by the resistance valve are crucial to the accuracy, reproducibility, and intercomparability of the test results. To date, little attention has been paid to this important issue. The details of the resistance valve are often missing in studies concerning various *in-vitro* test loops.

This study aims to quantitatively investigate the additional blood damage induced by the resistance valve and its relative importance compared with blood damage induced by the blood pump itself under different operating points, using CFD combined with hemolysis and platelet activation models. Idealized tubing models under a resistance valve with different degrees of valve opening were paired with a broad range of pump working conditions. Simulations of the tubings and the blood pumps were conducted in parallel with the same working conditions.

## 2 Materials and methods

### 2.1 Geometric models

#### 2.1.1 Idealized tubing models under resistance valve

**FIGURE 1:**
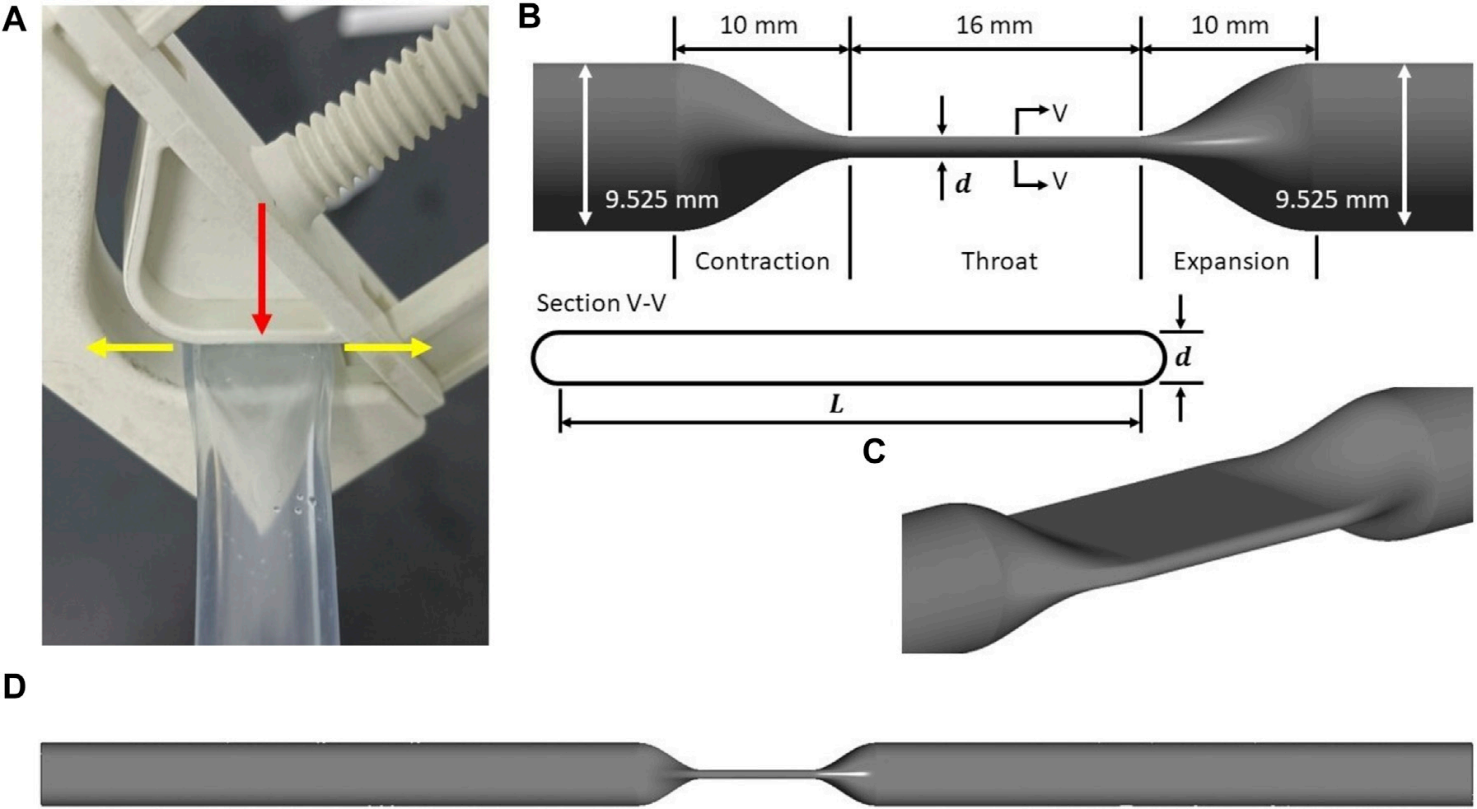
**(A)** A typical resistance valve (BioValve BV1000NW) with a V-groove, acting on the tubing with a side wall. Red arrow refers to the compression direction when the resistance valve is clamped, while yellow arrows refers to the extension direction when the tubing is compressed. Idealized tubing model: **(B)** schematic of the idealized throat, showing main design parameters and the cross-section (inner wall only); **(C)** isometric view of the idealized throat; **(D)** schematic of the idealized tubing model as the computational domain.

An idealized throat mimicking the effect of the resistance valve model was designed with straight round tubing at both ends (see Figure 1). The inner diameter ( D ) of the straight round tubing is 3/8 inch (9.525 mm), the standard tubing diameter for *in-vitro* blood damage testing (Wu et al., 2021). Figure 1A shows a typical resistance valve (BioValve BV1000NW, a variable flow control valve, Watson-Marlow Fluid Technology Group, Falmouth, Cornwall, UK), acting on the tubing (Tygon ND 100-65 ADF00028, Saint-Gobain Performance Plastics, Akron, OH, United States). The valve with a V-groove acts on the tubing with a sidewall. When compressed, the tubing stretches perpendicularly to the direction of compression. The idealized tubing model was constructed using Ansys SpaceClaim (Ansys Inc., Canonsburg, PA, United States), and only the inner wall was drawn. The idealized throat in Figure 1B is shown along with its cross-section. The length of the throat is 16 mm, as per the thickness of the BioValve BV1000NW. The circumference of the inner wall was assumed to be a constant of 29.92 mm (the original circumference of the tubing Tygon ND 100-65 ADF00028) during the compression. The cross-section was idealized as a pancake, with two parallel sides in the middle, and both ends closed with semicircles that are tangent to the parallel sides (see Figure 1C). The tubing model was extended at both ends by the length of 10*D* (95.25 mm). A contraction section and an expansion section were constructed upstream and downstream of the throat to allow for smooth transitions between the throat and the parts of tubing that were not affected by the compression. The total length of the idealized tubing model, as shown in Figure 1D, is 226.5 mm.

#### 2.1.2 Blood pump models

Three centrifugal blood pumps were considered. The first is the FDA benchmark blood pump which is a extracorporeal pump with mechanical bearings and extensive experimental results of flow field, pressure head and hemolysis available. Nonetheless, its hemolysis level is known very high and not a true representation of modern-day blood pumps. Thus, to evaluate the hemolysis effect of the resistance valve on commercial blood pumps, two intracorporeal maglev commercial pumps, CH-VAD (CH Biomedical, Suzhou, China) and HM3 (HM3) (Thoratec Corporation, Pleasanton, CA, United States) were also included.

**FIGURE 2:**
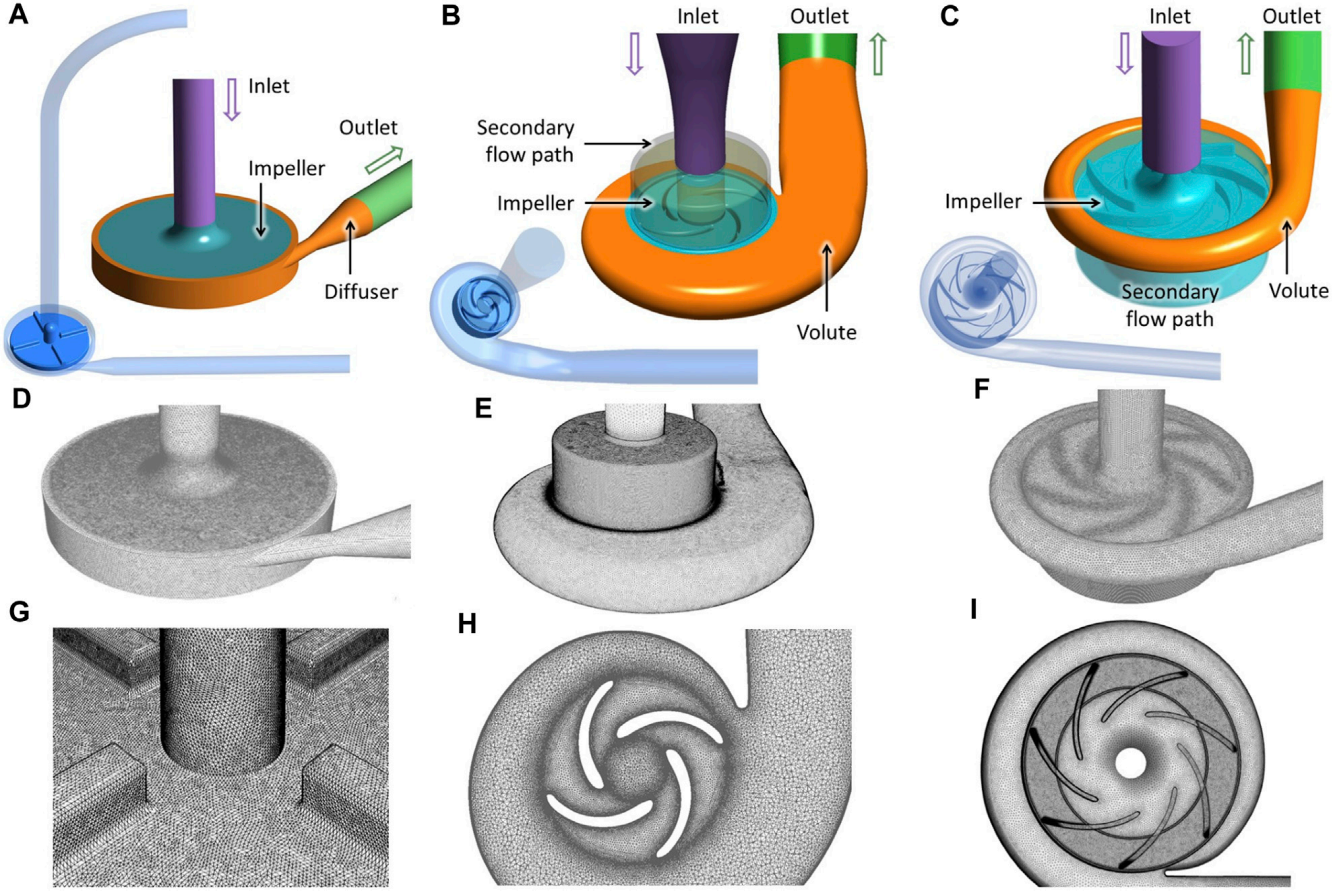
Geometry **(A–C)**, grid **(D–F)**, and details of the “fine” mesh (cf. Table 1) at impeller region **(G–I)** of the three blood pumps employed in this study: the FDA benchmark pump, the HeartMate 3, and the CH-VAD (from left to right).

The FDA blood pump has simple geometric features, with 4 filleted blades positioned orthogonally on the rotor (see Figure 2A). The geometry of the HM3 was reconstructed from computed tomography (CT) scans by Wiegmann et al. (Wiegmann et al., 2018), and consists of an inflow cannula, a shrouded centrifugal impeller, a lower housing and an upper housing (see Figure 2B). The CH-VAD is a centrifugal pump featuring a fully maglev impeller and an integrated inflow cannula. A secondary flow path surrounds the impeller in the suspension gap between the rotor and lower housing. Geometry of the CH-VAD flow path shown in Figure 2C was extracted from CAD files.

### 2.2 CFD operating points and analysis

#### 2.2.1 Operating points

Table 1 shows eight investigated operating points. Four working conditions of the FDA were selected ranging from low flow (2.5L/min) to high flow condition (6.0L/min), while for CH-VAD and HM3, conditions of 5L/min and 8L/min were studied. Each of the eight selected operating points was paired with a degree of valve opening, implemented through the variation of throat thickness *d*, which is also the diameter of the semicircles on both sides (cf. Figure 1D). Since the circumference of the inner wall was assumed to be a constant of 29.92 mm during the compression, the shape of the throat can be uniquely determined by a given throat thickness *d*. Thus, the length of the two parallel sides *L*, and *d* satisfy:

2L+πd=29.92mm (1)

**TABLE 1:** Operating points and design variations of throat.

Case #	Pump			Throat	
	Model	Flow rate (L/min)	Rotational speed (rpm)	d (mm)	L (mm)
1	FDA	2.5	2,500	0.644	13.95
2	FDA	2.5	3,500	0.475	14.22
3	FDA	4.5	3,500	0.745	13.79
4	FDA	6.0	3,500	1.031	13.34
5	HM3	5.0	6,000	1.376	12.80
6	HM3	8.0	7,700	1.800	12.13
7	CH-VAD	5.0	3,155	1.376	12.80
8	CH-VAD	8.0	3,830	1.800	12.13

As will be shown later in the results section, these operating points cover a broad range of pressure heads, from 90.7 to 340.3 mmHg.

The operating points listed in Table 1 were all studied using CFD for both the tubing models and the blood pumps. The pressure loss induced by other loop components was ignored, and the pressure head of the pump was assumed to be completely offset by the pressure drop of the idealized tubing model.

The FDA pump and the HeartMate 3 (Cases #1 through #6) were first simulated at the flow rates and rotational speeds listed in Table 1 to obtain their pressure heads and blood damage levels. Thus, the degree of valve opening of the tubing models was adjusted to match the pressure drop with the pressure head at the corresponding operating point of the blood pumps. The parameters of the throat, *d* and *L*, were iteratively adjusted through CFD computations, and fine-tuned to 3 decimal places (1 μm) to keep relative errors of the two pressures below 0.75%. The resulting geometric parameters of the throat are shown in Table 1. An in-house Python code was employed in combination with recorded journals in Ansys to automate the above iterative simulation workflows. It takes 5 to 8 iterative adjustments to get satisfactory parameters. Blood damage of the tubing model were then calculated after the geometric parameters were determined.

The CH-VAD cases (#7 and #8) followed a different process. To match the operating point of Case #7 with that of #5, and #8 with #6, in terms of flow rates and pressure heads, the rotational speed of the CH-VAD was iteratively adjusted through CFD computations, and fine-tuned to 5 rpm to keep relative errors of the pressure heads below 0.75%. Blood damage of the pump was then calculated after rotational speed was determined. Cases #7 and #5 shared the same tubing model, as did Cases #8 and #6.

#### 2.2.2 CFD analysis of the blood pumps

Following the recommendations of (Wu et al., 2022), full unstructured grids were generated for all pump models - the FDA blood pump, the HeartMate 3, and the CH-VAD - using Ansys Meshing (Ansys Inc., Canonsburg, PA, United States). Grid sensitivity analysis was performed, with three grids - “coarse”, “medium” and “fine” generated for the three pumps models, as shown in Table 1. For all models, flow rates of each operating point were imposed at the inlet. Ansys Fluent was used to perform the CFD computations. Turbulence was modeled using the SST k-ω model, and the SIMPLE method was employed to solve the incompressible N-S equations. Second-order implicit scheme and second-order upwind scheme were employed for time and spatial discretizations. All simulations of the three blood pumps were transient, and the sliding-mesh approach was used to couple the rotational and stationary frames. The “Pressure-outlet” boundary condition was imposed at the outlets of all models. For the FDA blood pump and Heartmate Ⅲ, each rotor rotation was resolved using 720 timesteps, with maximum 25 sub-iterations for each physical timestep, while for CH-VAD, each rotor rotation was resolved using 960 timesteps, as shown in Table 2. This ensured a Courant number close to 1. The blood was treated as a Newtonian fluid with a density of 1,055 kg/m^3^ and a viscosity of 0.0035 Pa∙s. Convergence criteria were set that the residuals of all equations drop below 10^-6^. On average, 10 impeller rotations were needed for main pump performance metrics to reached convergence. Another two rotations were taken to obtain the time-averaged pressure head and indices of blood damage.

**TABLE 2:** Grids and time resolution for CFD analysis of three pumps models.

Pump model	Grid number (million)	$y^{+}$	Time resolution (timesteps/rotation)	Courant number
FDA	19.50	≤0.63	720	≤0.73
Heartmate Ⅲ	25.06	≤0.56	720	≤0.93
CH-VAD	25.34	≤1.11	960	≤1.18

The value $y^{+}$ was averaged over all the pump walls, while the Courant number was volume-averaged. The table shows the maximum values of the two quantities of all conditions investigated for the respective pump model.

#### 2.2.3 CFD analysis of the idealized tubing models

**FIGURE 3:**
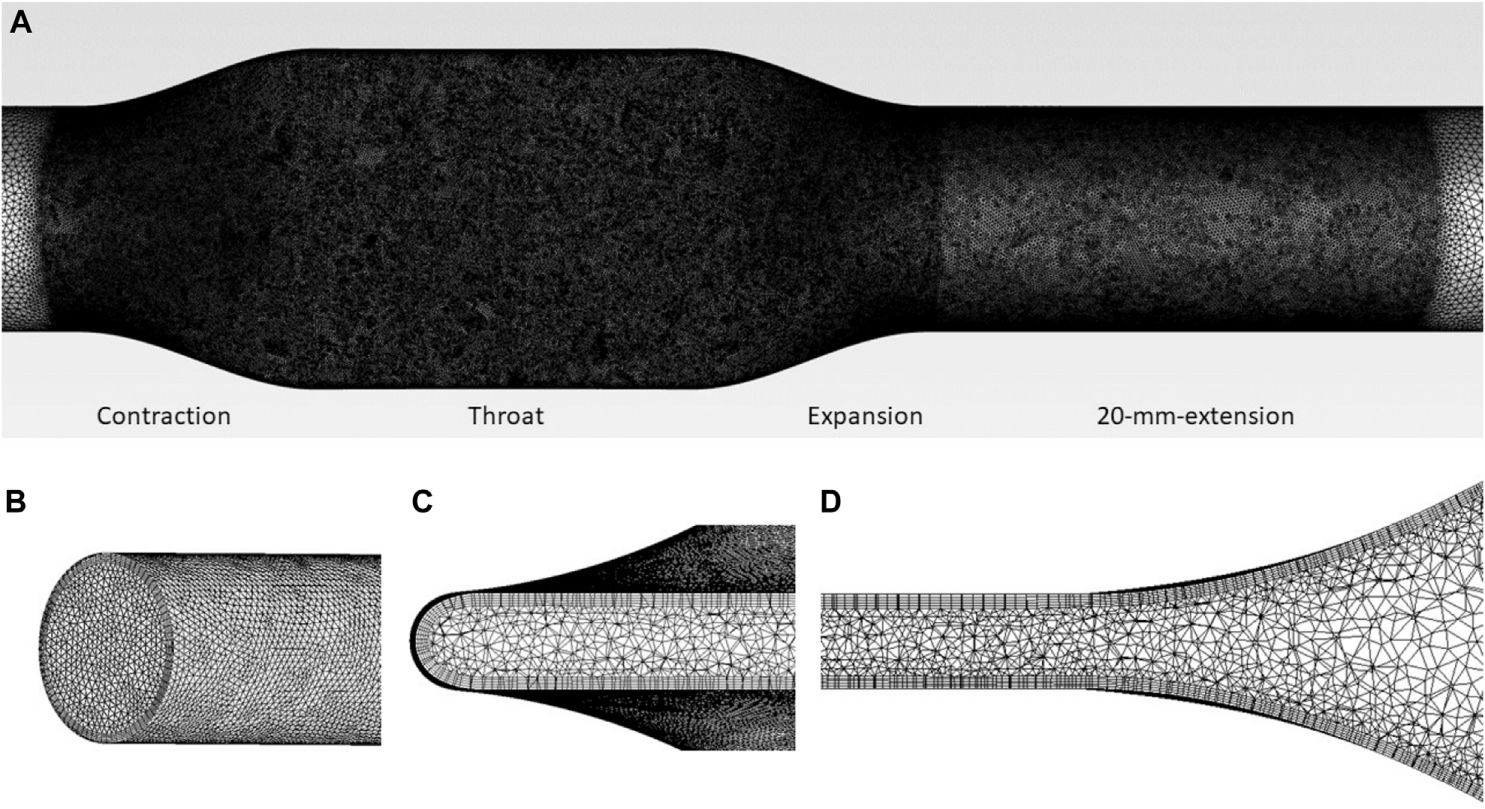
**(A)** Mesh of tubing model, showing the regions of mesh refinement. Mesh details are also shown at **(B)** inlet, **(C)** throat (cross-section), and **(D)** expansion (lengthwise-section) regions.

Unstructured tetrahedral grids shown in Figure 3 were generated using Ansys Meshing with 5 prism layers. The grids were refined at the contraction, throat, and expansion sections, as well as 20 mm downstream of expansion, as shown in Figure 3A. The distance of the first grid point from the wall, normalized using inner units, maintains y^+^< 1.5, such that the near-wall region is sufficiently resolved. For the tubing model with the same flow rate, the narrowed the tubing throat, the higher the Reynolds number will be. Thus, for a smaller ‘d’, the near wall mesh will be more critical compared with larger ‘d’ geometries and cases. Therefore, Grid sensitivity study was conducted for case 2 (with a narrowest throat of d = 0.475 mm), with three grids of 6.04 million, 13.23 million, and 26.86 million respectively. CFD simulations were performed using the same computational setup and material properties as the pump models. For each operating point, the pre-set flow rate was imposed at the inlet of the idealized tubing model. Convergence criteria were set that the residuals of all equations, except continuity, drop below 10^-6^.

#### 2.2.4 Hemolysis and platelet activation predictions

Power-law models were employed in this study to calculate indices of hemolysis and platelet activation. Power-law models relate blood damage to stress *τ* and exposure time *t* through a power-law relationship as (Heuser and Opitz, 1980; Song et al., 2003; Zhang et al., 2011; Ding et al., 2015)

$$D=C\tau^{\alpha }t^{\beta },$$ (2)

where D represent either hemolysis index in percentage (HI (%)) or platelet activation index (PAI), *C*, α and *β* are empirical constants, *τ* is effective stress and calculated according to the formulation of energy dissipation stress (Wu et al., 2019a):

$$\tau =\sqrt{\varepsilon \mu \rho }.$$ (3)

where ε is total energy dissipation rate, and has a unit of $J/\left(\text{kg}∙s\right)$ , representing the power loss per unit volume and unit time. ε was taken as the sum of resolved dissipation $\varepsilon_{vis}$ and modeled dissipation $\varepsilon_{turb}$ . Please refer to (Wu et al., 2019a; Wu et al., 2019b) for the definition and calculation of these two variables. Eq. 2 was solved through a Eulerian scalar transport approach (Garon and Farinas, 2004). Let

$$D_{I}=D^{\frac{1}{\beta }}=C^{\frac{1}{\beta }}\tau_{eff}^{\frac{\alpha }{\beta }}t,$$ (4)

where D represent either hemolysis index (HI (%)) or platelet activation index (PAI); $\tau_{eff}$ is scalar effective stress.

Then

$$\frac{{dD}_{I}}{dt}=\left(\frac{\partial }{\partial t}+\bar{u}\cdot \nabla \right)D_{I}=C^{\frac{1}{\beta }}\tau_{eff}^{\frac{\alpha }{\beta }}=\sigma ,$$ (5)

where σ represents source term for the prediction of blood damage. Ignore the unsteady effects and take the volume integration on both sides of Eq. 5, then

$$\int_{V}\left(\bar{u}∙\nabla \right)D_{I}dV=\int_{V}\left(\bar{u}∙\bar{n}\right)D_{I}dS=QD_{I}=\int_{V}\sigma dV=S$$ (6)

We defined S as the volume integral of the effective stress. Blood damage at the outlet of the computational domain could be obtained as

$$D={\left(D_{I}\right)}^{\beta }={\left(\frac{1}{Q}S\right)}^{\beta }={CS}^{\beta }$$ (7)

where $C=1/Q^{\beta }$ . Blood damage computed according to Eq. 7 was a rough estimate of blood damage since unsteady term was neglected.

We define $S_{p}$ and $S_{v}$ as the S for pump and valve respectively. To account for stress-history, the S for a single-pass through the pump and the valve can be computed as the summation of $S_{p}$ and $S_{v}$ . Then, blood damage can be computed as follows:

$$D_{p+v}=C{\left(S_{p}+S_{v}\right)}^{\beta }.$$ (8)

The blood damage of pump alone without resistance valve, and the additional hemolysis induced by the resistance can be obtained respectively as:

$$D_{p}=C{S_{p}}^{\beta },D_{v}=C\left[{\left(S_{p}+S_{v}\right)}^{\beta }-{S_{p}}^{\beta }\right]$$ (9)

Then, the valve-to-pump ratio R can be computed as:

$$R=\frac{D_{v}}{D_{p}}=\frac{{\left(S_{p}+S_{v}\right)}^{\beta }}{{S_{p}}^{\beta }}-1.$$ (10)

Eq. 10 also applies to the case of multi-pass experiment of blood tests. The valve-to-pump ratio of HI (%) and PAI are then defined as $R_{HI}$ and $R_{PAI}$ respectively. We also introduce $\eta_{HI,p}$ and $\eta_{HI,p+v}$ to represent the ratio of ${HI\;\left(\%\right)}_{p}$ (pump-only) and ${HI\;\left(\%\right)}_{p+v}$ (pump + valve) for two arbitrary cases A&B:

$$\eta_{HI,p}=\frac{{HI\;\left(\%\right)}_{p,A}}{{HI\;\left(\%\right)}_{p,B}},\eta_{HI,p+v}=\frac{{HI\;\left(\%\right)}_{p+v,A}}{{HI\;\left(\%\right)}_{p+v,B}}$$ (11)

$\eta_{PAI,p}$ and $\eta_{PAI,p+v}$ are likewise introduced for the PAI.

To reduce uncertainty, two commonly used sets of coefficients, the HO constants (Heuser and Opitz, 1980; Song et al., 2003) and the TZ constants (Zhang et al., 2011) were employed to compute hemolysis. $R_{HI}$ was then obtained by averaging the two ratios using the two sets of constants, to reduce the uncertainty. Table 3 lists the three sets of empirical constants employed in this study. Predictions of blood damage started after flow simulations had converged, with all flow variables frozen.

**TABLE 3:** Empirical constants of the power-law models for blood damage.

Model	C	α	β
Hemolysis (HO)	$1.800\times 10^{-4}$	1.9910	0.7650
Hemolysis (TZ)	$1.228\times 10^{-5}$	1.9918	0.6606
PAI (Ding)	$4.08\times 10^{-5}$	1.56	0.8

The HO, constants, originally derived from (Heuser and Opitz, 1980) by (Song et al., 2003). TZ, constants, proposed by (Zhang et al., 2011). PAI, platelet activation index, coefficients derived by (Ding et al., 2015).

## 3 Results

### 3.1 Validation of CFD simulations

#### 3.1.1 Pump models

Table 4 shows the predicted pressure head, hemolysis index (HI) and platelet activation index (PAI) for the three pump models in comparison with the results predicted with the respective fine mesh. Grid convergence was well achieved for all the three pump models. The “fine” grids were chosen to minimize the error of predicted metrics.

**TABLE 4:** Results of grid sensitivity analysis for the pump model.

Pump Model	Mesh Resolution	Cells (× $10^{6}$ )	Nr	P (mmHg)	Error of P	Error of HI	Error of PAI
FDA	Coarse	4.17	9	215.2	9.16%	6.99%	2.19%
6L/min	Medium	8.26	11	232.0	2.07%	2.98%	1.25%
3500rpm	Fine	19.50	11	236.9	—	—	—
Heartmate Ⅲ	Coarse	7.45	8	89.5	1.32%	8.87%	6.95%
5L/min	Medium	14.73	10	89.7	1.11%	0.27%	0.16%
6000rpm	Fine	25.06	10	90.7	—	—	—
CHVAD	Coarse	7.13	8	92.0	0.65%	0.49%	2.03%
5L/min	Medium	14.05	10	91.5	0.11%	0.26%	1.45%
3155rpm	Fine	25.34	10	91.4	—	—	—

Nr, number of grid points across the boundary layer; P, predicted pressure drop; Error of P, defined as $\left|P-\right.P_{0}\left|/\right.P_{0}$ , where $P_{0}$ is the pressure drop predicted with the fine mesh; Error of HI and PAI, errors of HI and PAI predictions, compared with the results of the fine mesh. The Error of HI was the average error of the HO and TZ constants.

Table 5 shows the pressure heads of all three pump models at various operating points, in comparison with experimental results (Thoratec Corporation, 2010; Malinauskas et al., 2017; Berk et al., 2019). CFD predictions agree reasonably well with the experimental data, with average error within 5%. It can also be observed that a broad range of pressure heads was covered, from 90.7 to 340.3 mmHg. Specifically, pressure heads were 90.7 and 110.8 mmHg (hypertension) for HeartMate 3, 91.4 and 111.4 mmHg (hypertension) for CH-VAD, and in the range of 167.5–340.3 mmHg for FDA benchmark pump. This corresponds well with the scenarios of evaluation and clinical application for intracorporeal and extracorporeal blood pumps.

**TABLE 5:** Pressure heads of the pump models at various operating points, in comparison with mean experimental results (Thoratec Corporation, 2010; Malinauskas et al., 2017; Berk et al., 2019).

Model		CFD results (P, mmHg)	Experiment results (P, mmHg)	Error (%)
FDA	2.5L/min,2500rpm	167.5	170	1.47
	2.5L/min,3500rpm	340.3	355	4.14
	4.5L/min,3500rpm	309.7	325	4.71
	6L/min,3500rpm	236.9	270	12.26
HeartmateⅢ	5L/min,6000rpm	90.7	90	0.78
	8L/min,7700rpm	110.8	105	5.52
CH-VAD	5L/min, 3200rpm	99.5	102.6	3.02
	8L/min, 3600rpm	94.9	103	7.86

**FIGURE 4:**
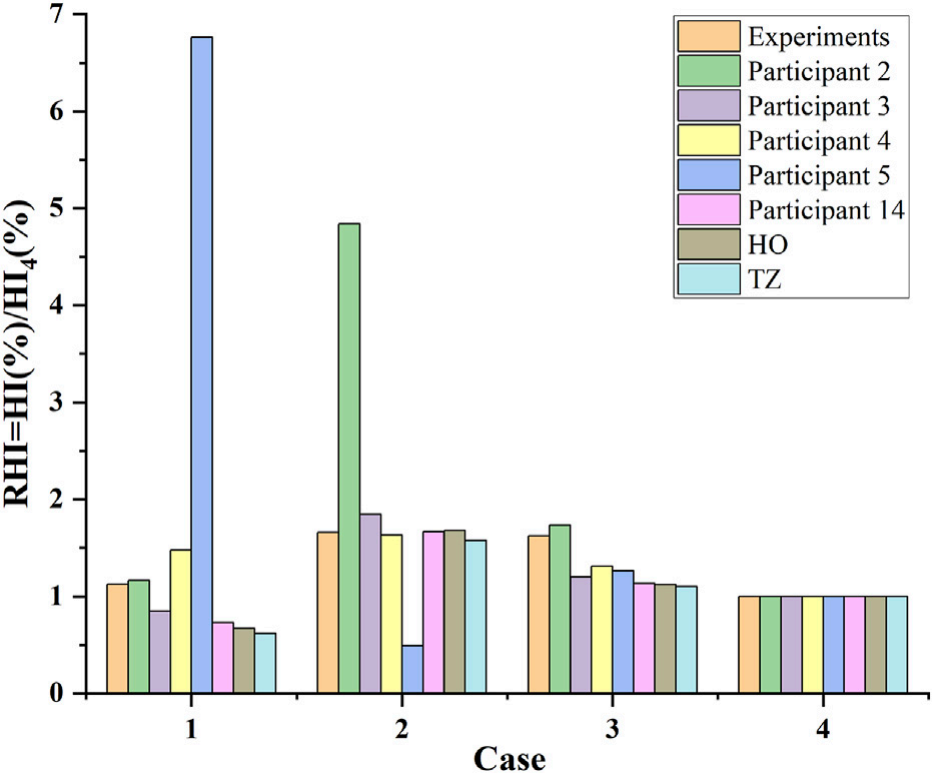
CFD hemolysis predictions of the FDA blood pump in this study (the “HO” and “TZ”), compared with CFD hemolysis predictions in the FDA round robin study (participants 2, 3, 4, 5 and 14, cf. (Ponnaluri et al., 2022)) and experimental measurements (mean value) for the relative hemolysis index (RHI) calculated in terms of the hemolysis index (HI (%)), normalized using the respective HI (%) of case 4 (cf. Table 1).

Figure 4 shows the predicted relative hemolysis index (RHI), calculated in terms of the hemolysis index (HI (%)), normalized using the respective HI (%) of case 4 (6.0 L/min and 3500 RPM, cf. Table 1), in comparison with CFD hemolysis predictions in the FDA round robin study (Ponnaluri et al., 2022) and experimental results. Table 6 shows the correlation coefficients between predicted hemolysis of the FDA blood pump and experimental hemolysis index. The correlation coefficient of the results obtained using the HO and TZ models are comparable to the first league of the CFD results in the FDA round robin study (Ponnaluri et al., 2022). Thus, the credibility of the simulation results of the pump models are proved.

**TABLE 6:** Correlation coefficients between predicted hemolysis of the FDA blood pump and experimental hemolysis index.

	Participant 2	Participant 3	Participant 4	Participant 5	Participant 14	HO	TZ
Correlation Coefficient	0.7346	0.7821	0.6188	−0.4665	0.7549	0.7415	0.7284

#### 3.1.2 Tubing models

Table 7 shows the results of grid sensitivity analysis for the Case #2 of the tubing model. Pressure head and hemolysis index were compared to the results predicted with the fine mesh. As shown in Table 4, the results using the medium mesh was sufficiently resolved, with the error of pressure head within 2%, and the error of HI and PAI below 1%. For lower computational costs, the medium mesh was employed for Case #2, and other variants of the tubing model were meshed with similar settings and grid sizes.

**TABLE 7:** Results of grid sensitivity analysis for the tubing model of case 2.

Mesh Resolution	Cells (× $10^{6}$ )	P (mmHg)	Error of P	Error of HI	Error of PAI
Coarse	6.04	315.18	7.19%	6.32%	13.64%
Medium	13.23	334.46	**1.51%**	**0.64%**	**0.23%**
Fine	26.86	339.60	**—**	**—**	**—**

P, predicted pressure drop; Error of P, defined as $\left|P-\right.P_{0}\left|/\right.P_{0}$ , where $P_{0}$ is the pressure drop predicted with the fine mesh; Error of HI and PAI, errors of HI and PAI predictions, compared with the results of the fine mesh. The Error of HI was the average error of the HO and TZ constants.

The bold numbers indicate that the error between the solution results of the medium grid and the fine grid is very small, less than 2 percent.

### 3.2 Hemolysis and platelet activation

**FIGURE 5:**
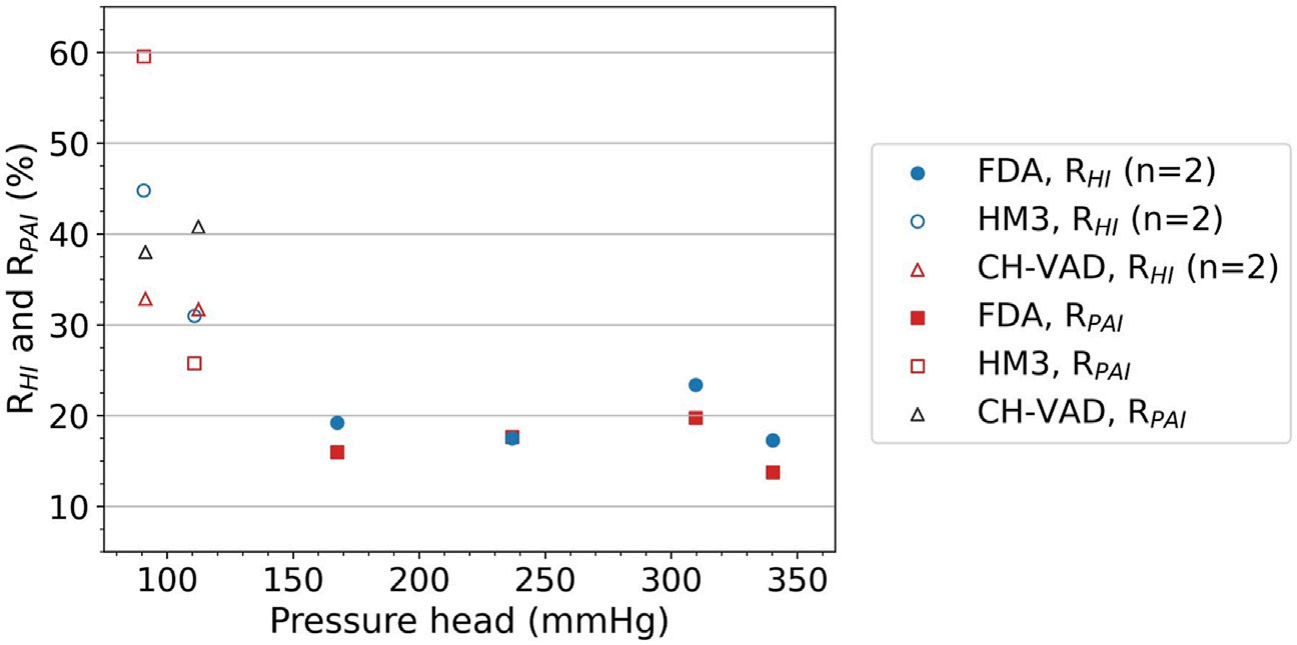
$R_{HI}$ and $R_{PAI}$ , valve-to-pump ratios of HI (%, 2 models, mean ± SD) and PAI (%). FDA, FDA benchmark pump; HM3, HeartMate 3.

Figure 5 shows the $R_{HI}$ and $R_{PAI}$ , valve-to-pump ratios of HI and PAI. For hemolysis, the ratio for each case was calculated by averaging the two ratios separately derived from the two sets of empirical constants (cf. Table 2), presented with standard deviations (SD). For the FDA blood pump, the results are relatively low, with $R_{HI}$ around 20% (17.3%–23.4%) and $R_{PAI}$ in the range of 13.8%–19.7%. Ratios for HeartMate 3 are generally higher, with $R_{HI}$ at 44.8% and 31.0%, and $R_{PAI}$ at 59.6% and 25.8%. As of the CH-VAD, all ratios are in a narrow range of 32.9%–40.8%. Among them, about 17% of $R_{HI}$ and 14% of $R_{PAI}$ were obtained in Case#2, while about 45% of $R_{HI}$ and 60% of $R_{PAI}$ were obtained in Case#5. While the CH-VAD shows a more stable trend, the differences of the ratios for HeartMate 3 between the two investigated operating points are much greater than the differences of the four operating points of the FDA pump.

In actual *in-vitro* testing, the blood damage levels measured are the result of the combined effects of the blood pump being tested and the loop components. The pump and loop contributions to blood damage are difficult to measure separately. A common approach is to compare new pump designs in parallel with benchmarked clinically approved device, so that the effects of loop components are presumably unimportant, to some extent. To provide more insight on this, blood damage caused by the pump and the valve is summed up in each of the Cases #5 through #8 (see Table 1) to reflect the joint effect of the pump and the loop. Both pumps used in these cases, the HeartMate 3 and the CH-VAD, are clinically approved devices. Cases #5 and #6 are comparable since they used the same pump model, as did Cases #7 and #8. And as described in the Methods section, Case #7 was paired with #5 at the same operating point, as were Cases #8 and #6. Relative changes between these cases in blood damage levels are shown in Table 8. Differences in hemolysis and platelet activation levels between different blood pumps or operating points can be attenuated by up to 38.2% and 43.1%, respectively.

**TABLE 8:** Relative changes in blood damage levels between cases.

Cases A, B	Commonality	HI			PAI		
		$\eta_{HI,p}$	$\eta_{HI,p+v}$	Δ	$\eta_{PAI,p}$	$\eta_{PAI,p+v}$	Δ
#6, #5	Pump, HM3	1.714	1.544	−23.8%	2.356	1.857	−36.8%
#8, #7	Pump, CH-VAD	1.325	1.305	−6.3%	1.106	1.128	21.0%
#7, #5	5L/min, 91 mmHg	1.200	1.124	−38.2%	1.456	1.260	−43.1%
#8, #6	8L/min, 111 mmHg	0.927	0.949	−29.8%	0.683	0.765	−25.8%

Δ , defined as $\frac{\left(\eta_{HI,p+v}-1\right)}{\left(\eta_{HI,p}-1\right)}-1$ or $\frac{\left(\eta_{PAI,p+v}-1\right)}{\left(\eta_{PAI,p}-1\right)}-1$

## 4 Discussion

Blood damage and their complications are major concerns during the clinical application of blood pumps. *In-vitro* testing has been employed as the key procedure to evaluate the hemocompatibility of blood pumps. Numerical modeling plays an increasingly important role in the evaluation of the hydrodynamic, hemolytic, and thrombogenic performance of blood pumps. Nonetheless, *in silico* simulations cannot replace *in-vitro* hemolysis testing yet. Thus, the credibility and accuracy of *in-vitro* blood circulatory testing are vital not only for the development and evaluation of blood pumps, but also for the development and validation of more accurate blood damage models. Hemolysis and platelet activation induced by loop components such as the resistance valve will affect the accuracy, reproducibility, and intercomparability of the results of *in-vitro* evaluations. Resistance valve is the critical loop component which offset nearly all the pressure head of the blood pumps. Nonetheless, little attention has been paid to the impact of the resistance valve on the results of these tests to date. Few specific recommendations concerning the resistance devices in the loop have been provided by industry standards or protocols, and occasional attempts to improve are not sufficiently justified.

**FIGURE 6:**
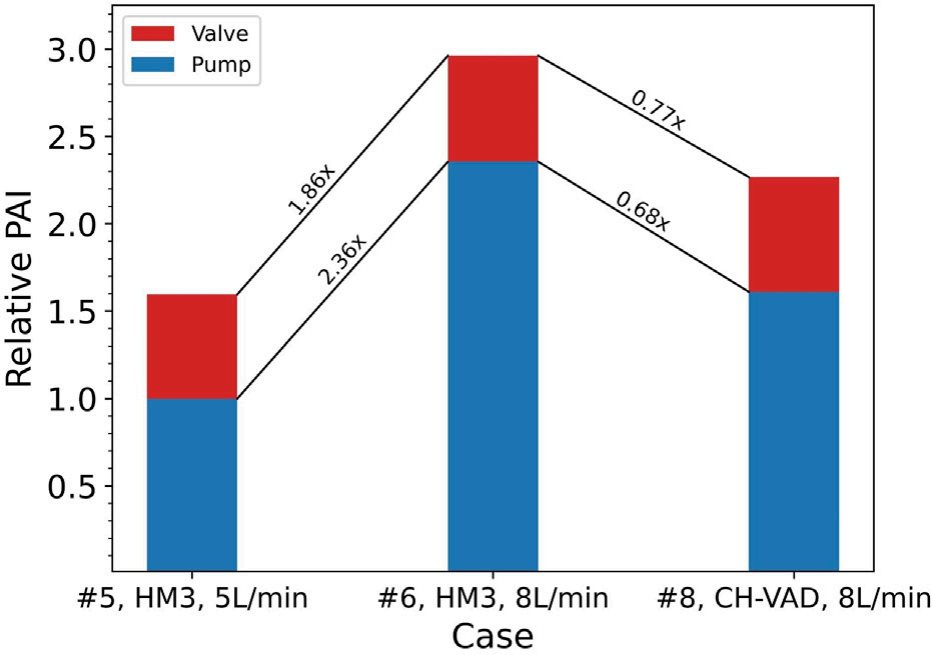
Ratios of relative changes between Cases #5, #6, and #8 in platelet activation levels of the pump itself and combined with the valve. Baseline: PAI of the HeartMate 3 (HM3) in Case #5 ( $7.836\times 10^{-4}\%$ ).

This study quantitatively investigated the hemolytic and platelet activation impact of the resistance valve during *in-vitro* blood damage testing, and found that blood damage induced by the resistance valve can be very high, becoming a significant source of error in the *in-vitro* test loops. Overall, hemolysis and platelet activation induced by a typical resistance valve are equivalent to 17%–45% and 14%–60%, respectively, of those induced by the pump itself. It is also worth noting that $R_{HI}$ was not a constant and varied greatly with flow rate and valve opening. There is no general rule concerning the variation trend of $R_{HI}$ and $R_{PAI}$ with pressure head (as shown in Figures 5, 6). The $R_{HI}$ for the FDA pump is much lower than that of the Heartmate 3 LVAD and the CH-VAD, and are possibly lower than most commercial blood pumps on market. Higher ratios indicate that the additional blood damage induced by the valve contributes more to overall test results. Thus, the influence of the resistance valve on the HeartMate 3 and the CH-VAD is much greater than that of the FDA blood pump. The reason is that the FDA blood pump was designed as a benchmark blood pump with simple features and very high hemolysis level (Malinauskas et al., 2017). It is not a good representation of commercial extracorporeal blood pumps in the 2020s, which have been optimized in almost every possible aspect to reduce blood damage. Nonetheless, 20% of the pump-induced hemolysis is a significant error for such an important benchmark model, and should be considered and addressed.

A common approach to address this issue is to validate the hemocompatibility of the investigated blood pump using a clinically approved device. This is done in an attempt to account for and eliminate the effects of loop components. However, this study suggests that such practices have limited effects. At the same operating point, the level of blood damage induced by different pumps varies, while the valve-induced damage remains largely the same. Figure 6 shows an example of such situations, where the CH-VAD generated 31% lower PAI than the HeartMate 3, but the overall PAI read from this hypothetical test is only 23% lower. Since blood damage induced by the resistance valve and other loop components are unavoidable and inseparable from pump-induced damage in real-world testing, “23%” is the inaccurate but only result. Direct comparison of results from different tests may underestimate changes in the blood damage levels of the pump itself.

In conclusion, the results of this study shed light on the impact of the blood circulating loop components on the accuracy of blood damage testing, contributing to a more accurate and credible evaluation method of the hemocompatibility of blood pumps.

One of the limitations of this study is the lack of experimental validations. It is not possible to perform experimental blood tests using a closed flow loop of just the valve, since a pump will be necessitated anyway to drive the flow and overcome the pressure drop of the valve and other loop components. For single-pass experiments using an open loop, it can be challenging to conduct such experiments under high flow rates at several liters per minute, mainly due to difficulties in sampling in a precisely and timely manner, which will possibly cause more errors and uncertainties to the results.

Since it is not viable to measure the blood damage induced by the valve and pump separately through experiment, hemolysis and platelet activation indices were obtained only through numerical simulations. However, modeling of blood damage still suffers from inaccuracy. The power-law models (cf. Eq. 2) employed to predict blood damage in this study were summarized from Couette flow fields where laminar shear stresses dominate. These models were also often criticized for being too simplistic, and failed in many circumstances, such as flows where normal stress dominates (Faghih and Sharp, 2020) and turbulent flows (Hund et al., 2010; Wu et al., 2019a; Wu et al., 2019b; Wu, 2022). Nonetheless, the power-law models are still the most widely used hemolysis models to date, and are widely used in the design, optimization and evaluation of blood pumps (Fraser et al., 2011; Wu, 2022). Normal stress was shown causing higher hemolysis compared with shear stress, which is particularly relevant for the resistance valve which creates a sudden contraction and expansion where normal stresses can be expected. Hemolysis in the resistance valve may be largely due to normal stresses. The level of hemolysis computed for considering shear stress and normal stress equally could underestimate an experimental hemolysis that may actually be due to normal stresses. Moreover, a Eulerian approach was employed to solve the power law model for blood damage. As Faghih et al. showed in (Faghih et al., 2023), as the erroneous treatment of exposure time in the Eulerian approach becomes more pronounced for accelerating flows such as that in the resistance valve.

The form of effective stress τ and how the effect of turbulence on blood damage is considered might also have significant effects on the prediction of hemolysis. In our previous studies (Wu et al., 2019a; Wu et al., 2019b), the power-law hemolysis models were improved through representing effective stress in terms of energy dissipation ε, which was derived from N-S equation through arithmetic manipulations. Despite its simple form, this new formulation of effective stress greatly improved the prediction of hemolysis for a wide range of flow conditions (Wu et al., 2019b). Better correlations with experimental data of blood damage were observed for a capillary tube, the FDA nozzle model and blood pump (Wu et al., 2019b). Thus, the energy dissipation stress was employed in this study and showed a good correlation with experimental data for the FDA blood pump (cf. section 3.1.1). Though the tubing model lacks experimental validation, the capillary tube and FDA nozzle model are very similar to the tubing model in the present study, in terms of form factors, flow regimes, etc. The second point to note is that HO and TZ constants (cf. Table 3) have quite different values of C. The predicted absolute levels of blood damage can be very different with each set of coefficients. Nonetheless, the trend of hemolysis is more important. High correlations can be observed between the results of both sets of constants and the experimental results, with correlation coefficients higher than 0.70, close to the correlation coefficient as reported in (32), and comparable to the first league of the CFD results in the FDA round robin study (Ponnaluri et al., 2022).

Attentions were also paid to increase the credibility and reduce uncertainties during simulations of blood damage. When calculating the valve-to-pump ratios, we took the average of the ratios obtained using the HO constants and TZ constants respectively to reduce the uncertainty. RANS turbulence prediction method was used in this study, which often fails to capture complex transitional and turbulent flows. This might bring errors to the prediction of hemolysis. Nonetheless, the SST k-ω turbulence model is commonly used for simulating flows in blood pumps, providing relatively accurate prediction of overall performance of various blood pumps (Wu, 2022). The predicted pressure head was in good agreement with experimental results. Therefore, the reliability and credibility of the numerical simulations can be proved. The predicted ratios of the valve-induced to pump-induced hemolysis and platelet activation provide a valuable reference to evaluate the impact of the resistance valve.

There are other types of multiple combinations of compressions (such as compression using the tip of the stem) could also achieve the same desired pressure drop. Nonetheless, we consider only one real scenario (compression using sidewall). We believe the conclusions based on this particular and real scenario are valuable and show the influence and error induced by the resistance valve on the credibility of *in-vitro* blood damage tests is important and should not be neglected. Furthermore, the tubing model under the resistance valve was idealized and considered rigid. The circumference across all tubing sections at all operating points was assumed to be constant, which might be inaccurate when clamped tightly. Other geometric parameters such as throat angles were not considered neither. A more realistic tubing model should be considered in future studies. This study only studied the impact of the resistance valve on the hemolysis and platelet activation results of the *in-vitro* loop. In future studies, additional blood damage caused by the resistance valve for a wider range of operating conditions, especially the low flow conditions, should be considered. Attention should be paid to the design and optimization of new resistance devices and other loop components with low stress concentration and reduced blood damage potential to reduce their impact on test results.

## Data Availability

The raw data supporting the conclusion of this article will be made available by the authors, without undue reservation.
